# Biosynthesis and Metabolism of Garlic Odor Compounds in Cultivated Chinese Chives (*Allium tuberosum*) and Wild Chinese Chives (*Allium hookeri*)

**DOI:** 10.3390/ijms23137013

**Published:** 2022-06-24

**Authors:** Shi-Wei Xia, Lin-Feng Hang, Siyad Ali, Xiao-Yu Xu, Yan-Jun Liu, Qian-Qian Yan, Qiu-Yu Luo, Yu Li, Li-Jing Lin, Huan-Xiu Li, Xiao-Ai Zhang, Lin-Kai Huang, Xiao Ma, Yun-Song Lai

**Affiliations:** 1 College of Horticulture, Sichuan Agricultural University, Chengdu 611130, China; xia-sw@stu.sicau.edu.cn (S.-W.X.); 15756269241@163.com (L.-F.H.); siyad.abdali1994@gmail.com (S.A.); 2020305038@stu.sicau.edu.cn (X.-Y.X.); liuyanjun@stu.sicau.edu.cn (Y.-J.L.); yqianqian@stu.sicau.edu.cn (Q.-Q.Y.); 202001479@stu.sicau.edu.cn (Q.-Y.L.); liyu@sicau.edu.cn (Y.L.); 14208@sicau.edu.cn (L.-J.L.); lihuanxiu@sicau.edu.cn (H.-X.L.); 13181@sicau.edu.cn (X.-A.Z.); 2 College of Grassland Science and Technology, Sichuan Agricultural University, Chengdu 611130, China; huanglinkai@sicau.edu.cn (L.-K.H.); maroar@126.com (X.M.)

**Keywords:** *Allium hookeri*, wild species, ASCO, allicin, RNA-seq, metabolome

## Abstract

Chinese chives is a popular herb vegetable and medicine in Asian countries. Southwest China is one of the centers of origin, and the mountainous areas in this region are rich in wild germplasm. In this study, we collected four samples of germplasm from different altitudes: a land race of cultivated Chinese chives (*Allium tuberosum*), wide-leaf chives and extra-wide-leaf chives (*Allium hookeri*), and ovoid-leaf chives (*Allium funckiaefolium*). Leaf metabolites were detected and compared between *A. tuberosum* and *A. hookeri*. A total of 158 differentially accumulated metabolites (DAM) were identified by Gas Chromatography—Mass Spectrometry (GC-MS) and Liquid Chromatography—Mass Spectrometry (LC-MS), among which there was a wide range of garlic odor compounds, free amino acids, and sugars. *A. hookeri* contains a higher content of fructose, garlic odor compounds, and amino acids than *A. tuberosum*, which is supported by the higher expression level of biosynthetic genes revealed by transcriptome analysis. *A. hookeri* accumulates the same garlic odor compound precursors that *A. tuberosum* does (mainly methiin and alliin). We isolated full-length gene sequences of *phytochelatin synthase* (*PCS*), *γ-glutamyltranspeptidases* (*GGT*), *flavin-containing monooxygenase* (*FMO*), and *alliinase* (*ALN*). These sequences showed closer relations in phylogenetic analysis between *A. hookeri* and *A. tuberosum* (with sequence identities ranging from 86% to 90%) than with *Allium cepa* or *Allium sativum* (which had a lower sequence identity ranging from 76% to 88%). Among these assayed genes, *ALN*, the critical gene controlling the conversion of odorless precursors into odor compounds, was undetected in leaves, bulbs, and roots of *A. tuberosum*, which could account for its weaker garlic smell. Moreover, we identified a distinct *FMO1* gene in extra-wide-leaf *A. hookeri* that is due to a CDS-deletion and frameshift mutation. These results above reveal the molecular and metabolomic basis of impressive strong odor in wild Chinese chives.

## 1. Introduction

Cultivated Chinese chives (*Allium tuberosum*) is a popular herb vegetable in Asia that is also known as garlic chives or Chinese leek. Like many other *Allium* plants, *A. tuberosum* is taken as a medicinal food due to its various phytochemicals and physiological effects [1,2]. *A. tuberosum* belongs to the *Allium* genus, which is one of the biggest genera in the family Liliaceae, containing more than 800 species [3,4]. There is a consensus in taxonomic study that the *Allium* genus should be divided into subgenera or species groups, although there is some debate over the specific taxonomic categories [5,6]. In China, the *Allium* genus is divided into the following nine species groups according to their botanic traits: Sect. *Porrum*, Sect. *Schoenoprasum*, Sect. *Cepa*, Sect. *Rhizirdium*, Sect. *Bromatoorrhiza*, Sect. *Haplostemon*, Sect. *Anguium*, Sect. *Molium*, and Sect. *Caloscordum*. *A. tuberosum* belongs to the group Sect. *Rhizirdium* [7]. Wild Chinese chives (e.g., *A. hookeri*) have a wide distribution in mountainous areas of southwest China and are called “wide-leaf chives” by locals. *A. hookeri* is a clump-forming herb that is also native to north India [8]. This species can be further classified into two subspecies according to their habits of withering in the winter, which is also supported by genotype analysis [9]. The subspecies that does not wither in the winter usually has extra-wide leaves.

*A. hookeri* is consumed as a wild vegetable or an herb medicine but is not commonly cultivated in China even though it has a similar appearance to the cultivated *A. tuberosum*. Commercial cultivars of *A. tuberosum* with wide leaves, which have emerged in recent years, are not *A. hookeri* nor did they originate from *A. hookeri* [10]. Recent studies have shed light on the exploration and utilization of these wild species due to their high nutrient value, stronger odor, and elite agronomic traits. *A. hookeri* has a totally different flavonoid compound and much higher amino acid contents and mineral nutrient elements than cultivated Chinese chives [11]. A methanol extract of *A. hookeri* roots can alleviate inflammation by suppressing inducible nitric oxide synthase (iNOS) and cyclooxygenase-2 (COX-2) expression in mouse cells [12]. A dietary supplementation of *A. hookeri* fresh roots and fermented root food improve antioxidant activities and growth in birds [13]. The long history of the domestication of *A. tuberosum* may have resulted in the loss of some traits that are considered valuable today.

Most *Allium* species, such as garlic (*A. sativum*), chives (*A. fistulosum*), and onion (*A. cepa*)—as well as the subject of this study, *A. tuberosum*—are well-known for their characteristic odor that is due to a sulfide, lipophilic, and volatile component usually referred to as garlic odor compound. The most common flavor precursors are S-allyl cysteine sulphoxide (Alliin, or ACSO), S-methyl cysteine sulphoxide (Methiin, or MCSO), and trans-S-1-propenyl cysteine sulphoxide (Isoalliin, or PeCSO) [14]. The abundance and composition of sulfoxides vary among *Allium* species; *A. tuberosum* contains all three precursors [15]. Flavor precursors themselves are odorless, and their immediate conversion to odor compounds relies on tissue damage that allows the sulfoxide stored in the cytosol to react with vacuolar enzyme alliinase (ALN) [16]. Today, the biosynthetic pathway framework of flavor precursors has become much clearer despite some of its details remaining controversial [17,18,19]. Several critical enzyme genes have been functionally identified [20]. They are *phytochelatin synthase* (*PCS*), *γ-glutamyltranspeptidases* (*GGT*), and *flavin-containing monooxygenase* (*FMO*) [21]. In contrast, the formation process of volatile compounds from precursors such as diallyl sulfide (DAS), diallyl disulfide (di-2-propenyl disulfide; DADS), and diallyl trisulfide (di-2-propenyltrisulfide; DATS) remains mysterious, which may be partially due to the very fast chemical reaction and subsequent difficulties in the extraction and detection of mid-stage compounds [19]. ALN, lachrymatory factor synthase (LFS), and spontaneous chemical processes are involved in the formation of downstream violate compounds [22].

Species in the genus *Allium* have an impressively large genome size with a range of 6.86 gigabase (Gb) to 30.87 Gb [23,24]. Due to this large genome size, there was no genome sequence release of *Allium* plants for a long time. Until now, only two *Allium* species have had a complete genome sequence reported and deposited into public databases; these are *A. sativum* (garlic) and *A. cepa* (onion) [25]. The assembled genome size of garlic is about 16 Gb, which is nearly the same size detected by flow cytometry. Because of the lack of high-quality reference genomes, omics studies of *Allium* plants lag behind those of many other vegetable crops even though the *Allium* genus contains many popular vegetables. Two transcriptomic studies of garlic chives provide a preliminary information of unigene sequences [26,27].

In this study, we compared the transcriptome and metabolome between cultivated Chinese chives (*A. tuberosum*) and wild Chinese chives (*A. hookeri*). The integrative network analysis explained the difference in garlic odor compounds. We isolated full-length gene sequences of critical enzymes that catalyze the formation of odor compound precursors, and analyzed the correlation of biosynthesis and metabolite in the odor compounds. The findings provide useful information on wild *Allium* species.

## 2. Results

### 2.1. Germplasm Curation and Nutrition Evaluation

**Figure 1:**
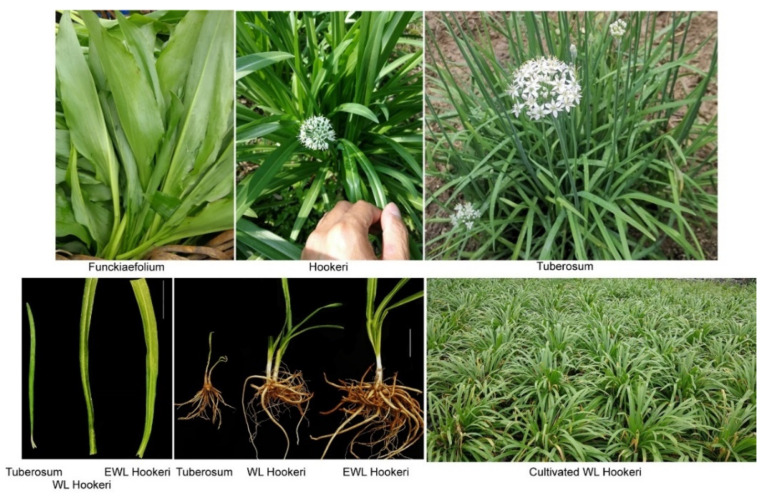
Pictures of Chinese chives and wild Chinese chives. The white vertical bar in the pictures indicate a scale of 5 cm. WL Hookeri, Wide-leaf *A. hookeri*; EWL Hookeri, extra-wide-leaf *A. hookeri*.

We collected one cultivated *A. tuberosum* plant and three wild materials from a mountainous area of Ya’an, Sichuan Province, China. Among the three wild species, one was *A. funckiaefolium*, which strictly grows in high-altitude areas (>2500 m) and is called “ovoid-leaf Chinese chives” by locals; and two were subspecies of *A. hookeri*, which are grown in a wide area below 2000 m and are called “wide-leaf Chinese chives” by locals (Figure 1). The two subspecies of *A. hookeri* are characterized by their wide leaves but differ from each other in leaf width. Wide-leaf *A. hookeri* (WL Hookeri) has a leaf width of 1.5 cm, while extra-wide-leaf *A. hookeri* (EWL Hookeri) has a leaf width of 2.4 cm. Both subspecies of *A. hookeri* have high levels of adaptability, whereas *A. funckiaefolium* cannot survive in low altitudes (Figure 1). As shown in Table 1, cultivated Chinese chives had a higher water content implying better edibility; however, *A. hookeri* had a stronger odor, and a higher content in soluble sugars and proteins. This difference might be a result of domestication and artificial selection.

### 2.2. Metabolite Comparison

Metabolites were compared between the leaves of *A. tuberosum* and WL Hookeri. For quality control and quality assurance in the detection of Gas Chromatography—Mass Spectrometry (GC-MS) and Liquid Chromatography—Mass Spectrometry (LC-MS), each sample was assayed six times (Figure S1). Both principal component analysis (PCA) and partial least squares-discriminate analysis (PLS-DA), a supervised PCA algorithm, showed well-clustered samples indicating high quality; the permutations plot indicated there was no overfitting in PLS-DA analysis (Figure S2).

**Figure 2:**
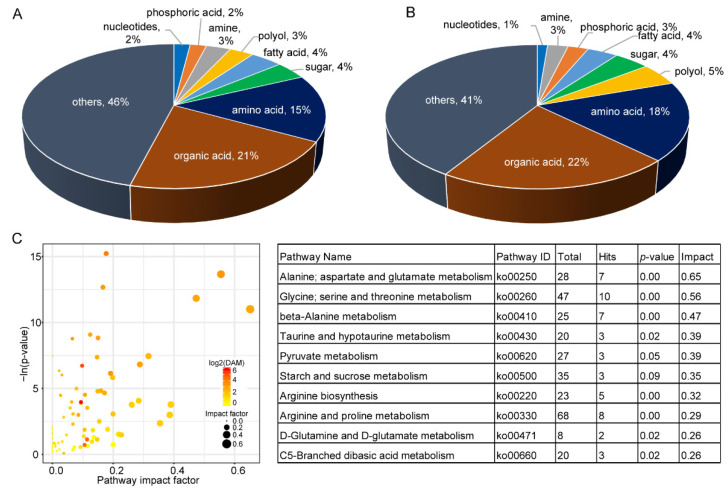
Metabolites identified by GC-MS and LC-MS. (**A**) Total metabolites. (**B**) Differentially accumulated metabolites (DAMs). (**C**) KEGG pathway enrichment analysis of DAMs. Left, overview of the enrichment analysis; right, top 10 enriched pathways (with the highest value of impact factor). Total, number of genes annotated in a pathway; Hit, number of genes hits in a pathway.

**Figure 3:**
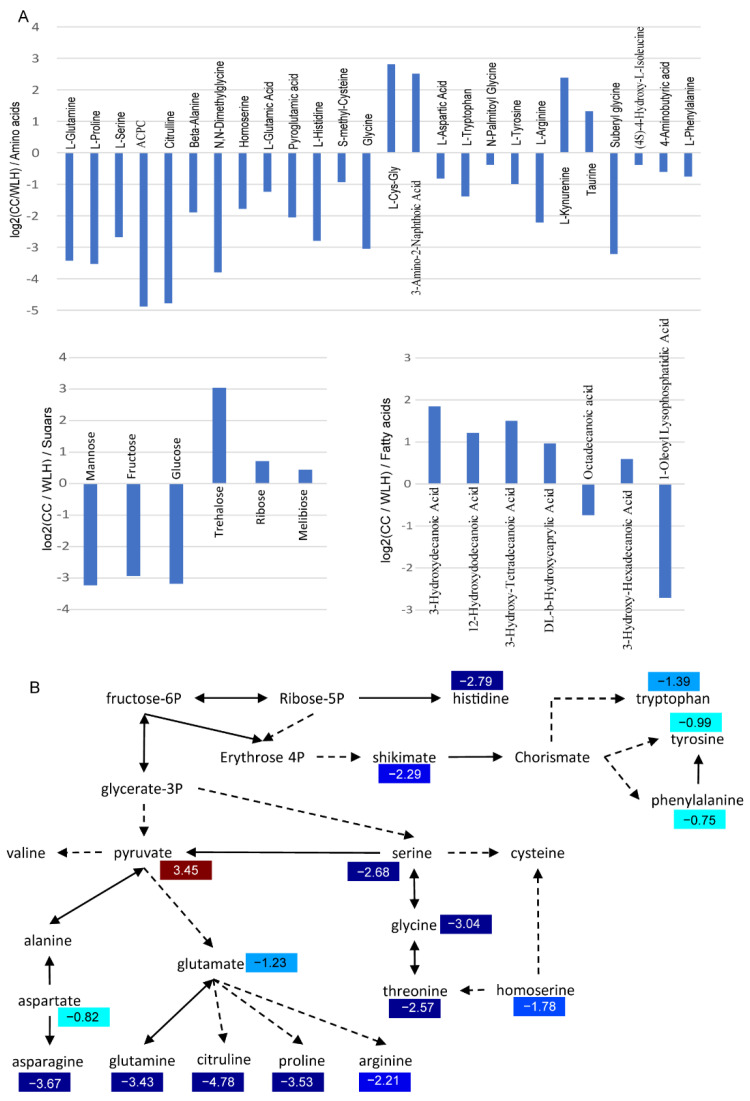
Differentially accumulated metabolites (DAMs) of amino acids, sugars, and fatty acids. (**A**) Change of metabolite indicated by Log2 (Fold change). ACPC, 1-Aminocyclopropanecarboxylic Acid. (**B**) DAMs in amino acids biosynthesis pathway (ko01230). Numbers in color indicate a heatmap of log2 (fold change). Fold change, change in *A. tuberosum* relative to *A*. *hookeri*.

A total of 69 and 245 chemical molecules were detected by GC-MS and LC-MS, respectively (Table S1). After discarding redundancies between the two methods, there were 304 components identified in leaves. Among the identified metabolites, there were 64 organic acids and 46 amino acids (Figure 2A). There were 177 significant differentially accumulated metabolites (DAMs) between *A. tuberosum* and WL Hookeri, whose composition was similar to that of the total metabolites (Figure 2B). However, the amino acids and polyols were exceptions, which took up 18% and 5%, respectively, in the DAMs. There were as many as 26 amino acids showing significant differences in content, most of which were much higher in WL Hookeri (Figure 3A). A Kyoto Encyclopedia of Genes and Genomes (KEGG) enrichment analysis of the DAMs indicated that more than half of the top 10 significant enriched pathways are amino acid metabolisms, e.g., alanine, glycine, pyruvate (Figure 2C). In the amino acid biosynthesis pathway (ko01230), all the DAMs showed higher contents in WL Hookeri, except for an intermediate pyruvate (Figure 3B). Notably, glutathione metabolism pathway as well as cysteine and methionine metabolisms pathway were significantly different; in particular, 19% of detected metabolites in the glutathione metabolism pathway were DAMs. These two pathways are known to be involved in the biosynthesis of garlic odor compounds.

Interestingly, the much higher accumulation of amino acid in WL Hookeri is accompanied by a much higher level of fructose, which is one of the substrates of amino acid biosynthesis (Figure 3B). Fructose in WL Hookeri is 7.7 times higher than in *A. tuberosum*, which might also account for the sweet taste of WL Hookeri. In addition to fructose, WL Hookeri accumulated more mannose and glucose, despite having less trehalose, ribose, and melibiose (Figure 3A). There are some interesting findings for other metabolites. Organic acids were the most common metabolites in the DAMs. There were seven fatty acids showing significant difference in content, and most of them had a lower abundance in WL Hookeri (Figure 3A).

### 2.3. Leaf Transcriptome Comparisons

The leaf transcriptomes of *A. tuberosum* and WL Hookeri were compared. A total of 39.9 Gb of clean data were obtained, and for each sample, the clean data were over 6.2 Gb. The percentage of high-quality bases (≥Q30) was over 92.5% for each sample (Table S2). We tried to assemble transcripts by first mapping to the garlic reference genome. However, the ratio of mapped reads was too low (<70%). Then, we carried out de novo assemblies without mapping to a reference genome. A total of 146,037 and 124,248 unigenes were obtained for *A. tuberosum* and WL Hookeri, respectively, and the N50 length of total unigenes was 1630 bp. We mapped clean reads against the unigene pool and the mapped ratio ranged from 72–77%. To further reduce low-quality unigenes, we blasted amino acid sequences against both the Swiss-Prot and the garlic genome, and the unigenes were discarded when the blast e-value was >1 × 10^−5^. As a result, we finally obtained 33,179 and 34,315 unigenes for *A. tuberosum* and WL Hookeri, respectively.

**Figure 4:**
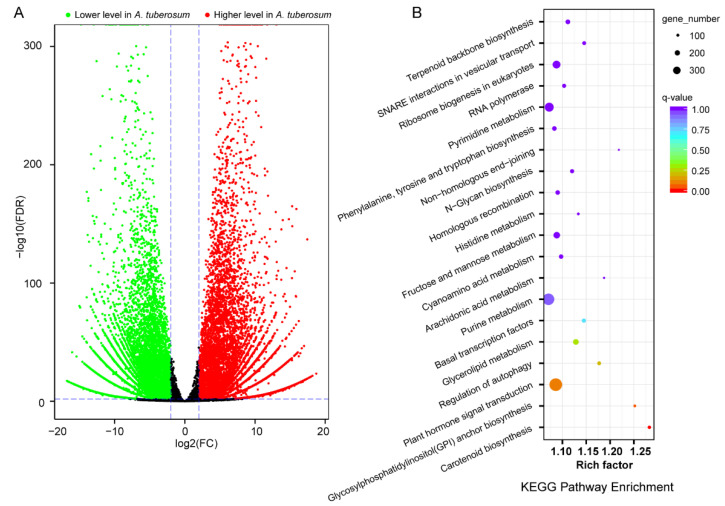
Profile of differentially expressed genes (DEGs) in comparative transcriptome analysis (*A. tuberosum* vs. *A*. *hookeri*.). (**A**) Volcano plot showing statistical significance of DEGs. (**B**) KEGG enrichment analysis.

We identified a total of 12,362 differentially expressed genes (DEGs), among which 4946 genes were higher and 7416 genes were lower in *A. tuberosum* (Figure 4). A KEGG enrichment analysis indicated that terpenoid backbone biosynthesis was the most significant pathway. Notably, there was significant difference in fructose and mannose metabolism. A Gene Ontology (GO) enrichment analysis indicated that photosynthesis was the most significant pathway (Table 2).

### 2.4. Gene Cloning of Garlic Odor Compounds

**Figure 5:**
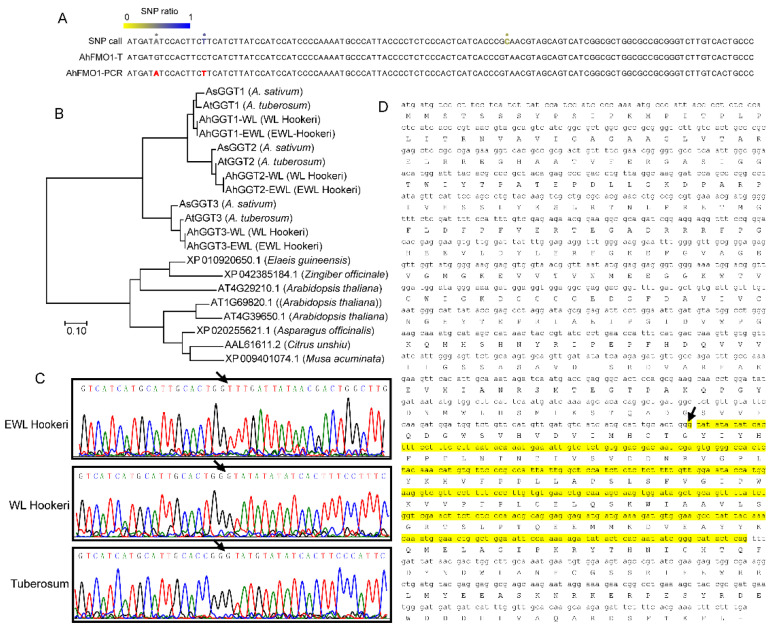
Gene sequential analysis of *GGTs* and *FMO1*. (**A**) A partial illustration of SNP calls that were obtained by mapping RNA-seq reads back to an assembled *AhFMO1-WL* transcript (*AhFMO1-T*). The ratio of SNP was calculated using RNA-seq reads. (**B**) Phylogenetic analysis of *GGTs* gene. (**C**,**D**) *FMO1* in EWL Hookeri lost a 310-bp CDS region (denoted in yellow color) which led to a frameshift and large deletion. The arrow indicates the start point of the frameshift.

Currently, sequence information of garlic odor compound biosynthetic genes is only known in garlic and onion. Here, we identified full-length sequences of genes related to the garlic odor compound by assembled unigenes; these are *GGT1*, *GGT2*, *GGT3*, *FMO1*, *PCS1*, and *ALN*. We manually assembled *AtGGT1* from four unigenes (BMK11240, BMK3806, BMK3807, and BMK94228) and *AhGGT3* from two unigenes (BMK121251 and BMK108624). The rest of the transcripts were determined from a single unigene sequence. To verify the gene sequence based on transcriptome assemblies, we sequenced PCR products of the above genes in *A. tuberosum*, WL Hookeri, and EWL Hookeri by Sanger sequencing. As a result, the final amino acid sequences of cloned genes show a very high level of identity (>99%) to the transcripts (Table 3). Some nucleotides with high divergency in RNA-seq reads were corrected by Sanger sequencing of PCR-products (Figure 5A; Table S4). Orthologue genes in WL Hookeri and EWL Hookeri show identical sequences.

In *Allium* plants, there are three *GGT* genes (*GGT1*, *GGT2*, and *GGT3*) as identified in *A. sativum*; in Arabidopsis, there are four *GGT* genes. Phylogenetic tree analysis indicated the three *GGT* genes in *Allium* are very different to *GGTs* in other plants, indicating the three *GGTs* were independently evolved after the formation of the *Allium* genus (Figure 5B). *GGT2* and *GGT3* were clustered, indicating a closer relation. *AhFMO1-EWL* encoded 317 amino acids in EWL Hookeri, which is much shorter than other sequences (Figure 5B). This is due to a deletion of 310 bp in mRNA, which resulted in 104 deletions of amino acids and a frameshift. This sequence variant is verified by PCR-based Sanger sequencing (Figure 5C,D).

### 2.5. Integrative Analysis of Garlic Odor Compound Biosynthesis

**Figure 6:**
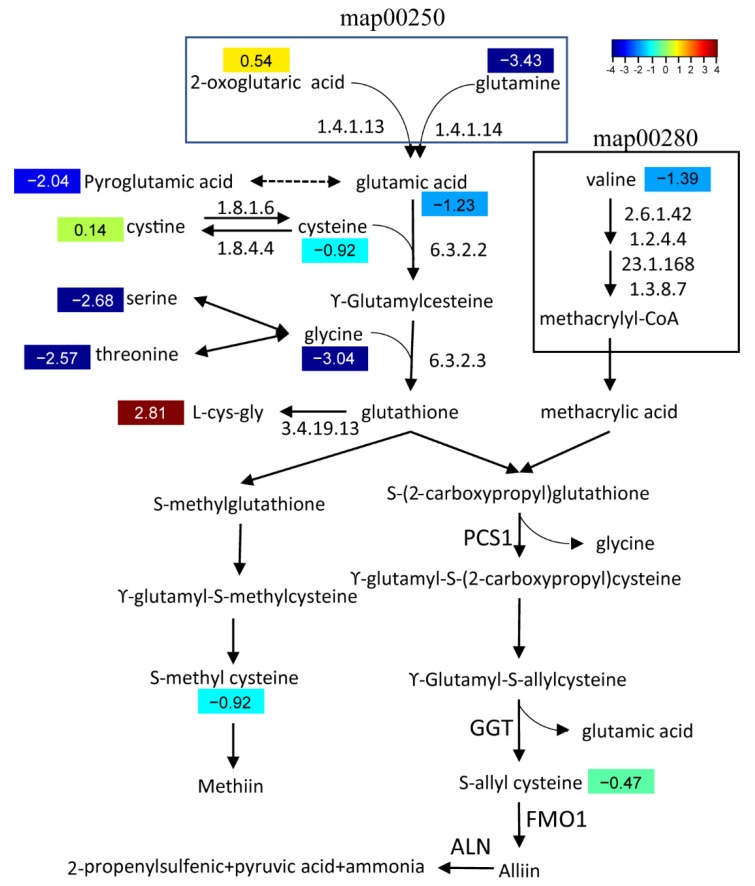
Biosynthetic pathway of a garlic odor compound. Pathways of valine, leucine, and isoleucine degradation (ko00280); alanine, aspartate, and glutamate metabolism (ko00250); cysteine and methionine metabolism (ko00270); and glutathione metabolism (ko00480) were involved in this pathway. Numbers in color indicate a heatmap of log2 (fold change). Fold change, change in *A. tuberosum* relative to *A*. *hookeri*.

Precursors of methiin and alliin, two major garlic odor compounds, were identified in both *A. tuberosum* and WL Hookeri. The content of alliin-precursor (*S*-allyl cysteine) and methiin-precursor (*S*-methyl cysteine) in WL Hookeri is 1.39 times and 1.9 times of those in *A. tuberosum*, respectively (Figure 6). No isoalliin- and cycloalliin-related compounds were identified. Nearly all the substrates in the biosynthetic pathway had a higher abundance in WL Hookeri, except for L-cys-gly. Especially of note, the content of glutamine in WL Hookeri is 3.6 times of that in *A. tuberosum*. Cysteine provides the critical sulfur in garlic odor compounds, which has a content 1.9 times the amount in WL Hookeri than in *A. tuberosum*. Lower substrate metabolites and stronger consumption by L-cys-gly together led to less accumulation of garlic odor compounds in *A. tuberosum*.

**Figure 7:**
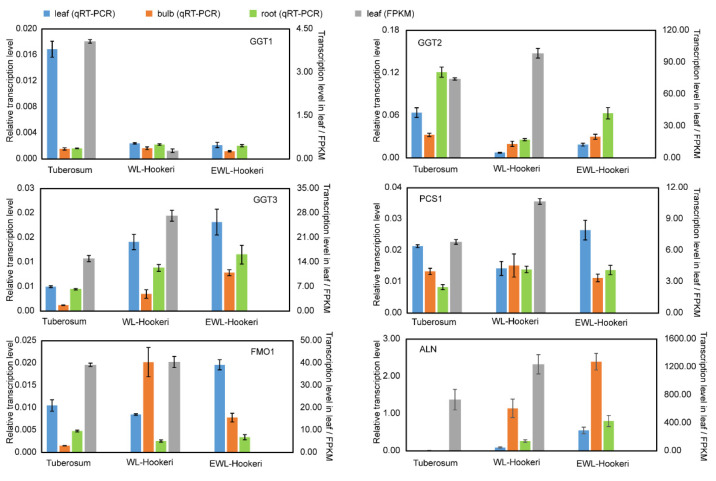
Transcription levels of *GGT1*, *GGT2*, *GGT3*, *PCS1*, *FMO1*, and *ALN* detected by qRT-PCR and RNA-seq counts. Left *y*-axis, relative transcription level modified by Actin in qRT-PCR; right *y*-axis, transcription level quantified by fragments per kilobase of transcript per million mapped fragments (FPKM). The bar represents SE of the mean.

We then compared the transcription level of structural genes in the biosynthetic pathway of the garlic odor compound precursor. Some DEGs were found at steps annotated by KEGG (Figure 6), but there were multiple DEGs for each step and they showed divergent change when compared between *A. tuberosum* and *A. hookeri*. For example, there were six DEGs functionally annotated to encode glutamate dehydrogenase (1.4.1.13) and five of them showed a significantly higher transcription level in Hookeri. For the six newly assembled transcript sequences, *GGT2*, *GGT3*, *PCS1*, and *ALN* all showed a higher transcription level in Hookeri while *GGT1* and *FMO1* did not (Figure 7). The gene transcription level quantified by reads of RNA-seq might be inaccurate in this study, because the unigene sequences were de novo assembled without a reference genome and some of the transcript sequences were manually assembled from multiple unigenes. Thus, we performed quantitative Real-Time PCR (qRT-PCR) to detect the relative transcription level (Figure 7). To select reliable reference genes for qRT-PCR, we validated the expression stability of candidate reference genes in different tissues by using BestKeeper (Table 4). As a result, *elongation factor-1α* (*EF-1α*) is the most stable reference gene, which is a ubiquitous and vastly conserved cytosolic protein in all eukaryotic organisms. The primers designed to quantify the six target genes in qRT-PCR showed high PCR-amplification efficiency (90–110%) (Figure S3). Basically, we get the same conclusion in comparative analysis of gene transcription levels by using qRT-PCR and RNA-seq. However, there are still some exceptions. For example, *GGT2* and *PCS1* had a higher FPKM value in WL-Hookeri which was quantified by reads number in RNA-seq, but a lower relative transcription level quantified in qRT-PCR. Among the three *GGTs*, *GGT2* is the major active gene which had the highest expression level in the three germplasms, which was followed by *GGT3*; *GGT1* seemed inactive in Hookeri. Interestingly, all six genes can be detected in leaves, bulbs, and roots, except for *ALN* in *A. tuberosum*. *ALN* was highly expressed in bulbs of Hookeri but seemed inactive in cultivated *A. tuberosum*, which might account for the weaker garlic odor smell in *A. tuberosum*.

## 3. Discussion

*Allium* plants are often taken as vegetable and medicine plants due to their rich biochemical components that humans can benefit from. Therefore, many researchers make an effort to dissect metabolite components and profile their response to environmental treatment. However, people usually focus on onion and garlic but not too much on other *Allium* crops, let alone *Allium* wild species, although there are some reports on *Allium hirtifolium* [28,29,30,31]. Here, we determined metabolites in *A. hookeri*, which is widely distributed in Asia. *A. hookeri* is also called wild Chinese chives, and therefore, we compared between *A. hookeri* and cultivated Chinese chives (*A. tuberosum*). A total of 341 metabolites were detected by targeted metabolome analysis based on GC-MS and LC-MS assays; these metabolites can be classified into nine groups. A recent extensive study of 30 garlic accessions using a similar LC-MS method reported 472 metabolites [32]. Apparently, there is a technical limitation to identify more metabolites. Higher resolution mass spectrometry and accurate mass tools must be developed to get a more comprehensive profile of *Allium* metabolome [33]. We observed a big difference in amino acid content between *A. tuberosum* and *A. hookeri*. There are as many as 28 DAMs of amino acids, and 86% of these differentially accumulated amino acids show much higher content in *A. hookeri*. The big difference is not only in term of the DAMs number but also the extent of changes. Content of 1-Aminocyclopropanecarboxylic acid in *A. hookeri* is incredibly 29.34 times of that in *A. tuberosum*. Such a tremendous difference is also observed in citrulline (27.40 times), dimethylglycine (13.91 times), asparagine (12.74 times), and proline (11.54 times). The content and composition of amino acids seems to vary in different *Allium* plants, which may be a result of a long history of cultivation or evolution [34]. A higher content of amino acids in wild species probably contributes to their higher tolerance to biotic and abiotic stress [35].

The composition of the flavor precursors and S-Alk (en) yl-L-Cysteine (SAC) derivatives varied among *Allium* species [15,36]. There is a characteristic ratio of different precursor components in particular species. In the onion species *Allium fistulosum* and *Allium schoenoprasum*, isoalliin is the predominant component, which accounts for more than 80% of the total contents; in contrast, the predominant component in *A. tuberosum* is methiin, which accounts for 80% of the total contents [15]. In this study, methiin and alliin, but not isoalliin, were detected by GC-MS and LS-MS, which is probably due to the contents of isoalliin being too low in the materials and its unstable chemical properties [37]. In a previous study, isoalliin accounted for only 7% of the total contents in *A. tuberosum* [15]. The ratio of methiin to alliin is about 6:1 in both *A. tuberosum* and *A. hookeri*, which indicates similar and specific ratios of precursor components in these two species. Together with the high identity of critical structural genes in precursor biosynthetic pathways, *A. tuberosum* and *A. hookeri* seem to have close phylogenetic relationships. However, *A. hookeri* contains more flavor precursors, about 1.5 times the amount in *A. tuberosum*. This indicates the domestication and cultivation of *A. tuberosum* may have caused a decrease in its garlic odor contents.

*Allium* plants are characterized by their flavors and odors. However, the biosynthetic pathway of these odor compounds is still under debate, and, in particular, the enzyme genes catalyzing each of them are not yet well identified [18]. For precursor alliin biosynthesis, three enzyme genes (*PCS*, *GGT*, and *FMO*) have been identified and characterized. Even so, these three structural genes have only been sequentially reported in garlic and onion. In this study, we identified these sequences in *A. tuberosum* and in two wild *A. hookeri* subspecies (Table S3). Unigene sequences from assemblies of RNA-seq were used as templated sequences. *GGTs* from *Allium* species formed an independent cluster in the phylogenetic analysis, indicating a special evolution of sulfite-containing odor compounds (Figure 5A). We detected a sequence variance of the *FMO1* gene, which catalyzed the last step in forming alliin. In WHL Hookeri, it is highly likely that *FMO1* lost its function due to a fatal deletion of 310 bp, which resulted in a deletion of the amino acid sequence and a frameshift. The definite influence of this sequence variance on *AhFMO1-EWL* function should be determined in the future.

We tried to use the garlic genome as a reference at first, but the mapping ratio was lower than 70%. Due to the large genome size of *Allium* plants, genome sequences have only been released for garlic and onion. Recently, whole genome sequence data for *A. hookeri* (SRR15098630) were released, but the sequence depth was too low and there was no gene annotation. Therefore, we carried out a *de novo* assembly of the unigene sequence without a reference genome. To overcome the disadvantage of not having a reference genome, we restrictively discarded low-quality unigenes by blasting against both the Swiss-Prot database and the garlic genome. We assembled transcripts of garlic odor compound-related genes from RNA-seq reads by a HISAT2 tool and manual operation. To check the reliability, we mapped the RNA-seq reads back to the assembled transcripts and found some nucleotides were divergent. This is probably due to a sequencing error or multiple allele sequences. To make a correct full-length sequence, PCR amplicons were sequenced again by Sanger sequencing. Besides number-limited errors of single nucleotides, only one 18-bp insertion of the *AtFMO1* gene was corrected. This indicates a high level of accuracy of unique assemblies in this study.

## 4. Conclusions

In this study, we compared metabolome and transcriptome between cultivated Chinese chives and Hookeri to reveal the metabolic and transcriptomic bases accounting for the stronger garlic smell and sweeter taste of wild Chinese chives. Hookeri accumulated amazing much more free amino acids, fructose, and glucose in leaves. In addition, higher level of garlic odor precursors was detected in Hookeri, which is due to the stronger metabolic flux from glutamine, valine, and cystine into the garlic odor biosynthetic pathway. *ALN* gene that controls the conversion from odorless precursors to volatile odor compounds was undetected in cultivated Chinese chives, which probably contribute to its weaker smell in addition. For the first time, we isolated full-length sequences encoding key enzymes of garlic odor biosynthetic pathway from wild *Allium* species. These sequences show closer phylogenetic relationship with those of cultivated Chinese chives than onion and garlic. A big size deletion was observed in the CDS region of *FMO1* in EWL hookeri, which result in serious deletion of the C-terminal region and subsequently possible function loss.5. Plant Materials and Methods.

### 4.1. Plant Materials

We collected all the garlic germplasm from a mountainous area in Ya’an, Sichuan Province, China at different altitudes. Land race *A. tuberosum* was collected at 980 m in a field, two materials of *A. hookeri* were collected at 1500 m in the wild, and *A. funckiaefolium* was collected at 2700 m in the wild. All the plants were transplanted in Sichuan Agricultural University’s Chongzhou planting base at an altitude of 560 m in May 2017. *A. funckiaefolium* failed to survive in the subsequent domestication, and the leaf organs were collected in the wild. *A. tuberosum* and *A. hookeri* leaves were collected in July 2018 for the following assay.

### 4.2. Physiological Indices

Vitamin C content in the leaves was determined by the 2,6-Dichloroindophenol sodium salt hydrate method in accordance with the national standard GB 5009.86-2016. Dry matters content was determined in accordance with the national standard GB 5009.3-2016. Reducing sugars were measured by the classic Fehling’s test in accordance with the national standard GB 5009.7-2016. Soluble protein content was determined by Coomassie Brilliant Blue in accordance with the standard NY/T 1205-2006 (with a modification of initiation mass).

### 4.3. GC-MS Assay

About 100 mg of leaf tissue for each sample was homogenized in liquid nitrogen by using a high-flux organization grinding apparatus. The powder was then suspended in 1.4 mL of precooled methanol in a tube and 60 μL of ribitol was added (0.2 mg/mL stock in methanol) as an internal quantitative standard. Metabolites were then extracted by ultrasound machine for 30 s. Then, 750 μL of precooled chloroform and 1.4 mL of deionized water were added (at 4 °C). They were then centrifuged for 10 min at 14,000 rpm (at 4 °C), and 1 mL of supernatant was transferred into a new centrifuge tube. Samples were blow-dried by vacuum concentration. Sixty μL of a 15 mg/mL methoxyamine pyridine solution was added and vortexed for 30 s and then reacted for 120 min at 37 °C. Sixty μL of a BSTFA reagent (containing 1% TMCS) was added into the mixture and reacted for 90 min at 37 °C. After a centrifuge at 12,000 rpm (at 4 °C) for 10 min, the supernatant was pipetted and stored for the following steps. For the quality control (QC) samples, 20 µL from each prepared sample extract was taken and mixed. QC samples were used to monitor analytical result deviations from these pool mixtures and compared to the errors caused by the analytical instrument itself. The rest of the samples were used for GC-MS detection.

Gas chromatography was performed on a HP-5MS capillary column (5% phenyl/95% methylpolysiloxane, 30 m × 250 μm i.d., 0.25 μm film thickness, Agilent J & W Scientific, Folsom, CA, USA) to separate the derivatives at a constant flow of 1 mL/min. An amount of 1 µL of the sample was injected in split mode by the autosampler and the split ratio was 20:1. The injection temperature was 280 °C; the interface was set to 150 °C, and the ion source was adjusted to 230 °C. The oven temperature was 60 °C for 2 min, ascending to 230 °C with an increase of 10 °C/min (300 °C for 5 min). The scanning model was 35–750 *m*/*z* (full scanning).

### 4.4. LC-MS Assay

About 100 mg of leaf tissue for each sample was used. The methods of homogenization, methanol extraction, and chloroform purification were the same for the GC-MS assay. The extract solution was dried by vacuum concentration and then dissolved with a 250 μL methanol aqueous solution (1:1, 4 °C). The sample solution was filtered by a 0.22 μm membrane and then was prepared for LC-MS detection. For the QC samples, 20 µL was taken from each prepared sample extract and mixed. The rest of the samples were used for LC-MS detection.

Chromatographic separation was accomplished in a Thermo Ultimate 3000 system (Thermo Fisher Scientific, Waltham, MA, USA) equipped with an ACQUITY UPLC^®^ HSS T3 (length 150 mm, inner diameter 2.1 mm, particle size 1.8 µm, Waters Co., Milford, MA, USA) column, which was maintained at 40 °C. The temperature of the autosampler was 8 °C. Gradient elution of the analytes was carried out with 0.1% formic acid in water (A) and 0.1% formic acid in acetonitrile (B) or 5 mM ammonium formate in water (C) and acetonitrile (D) at a flow rate of 0.25 mL/min. An amount of 2 μL of each sample was injected after equilibration. An increasing linear gradient of solvent B (*v*/*v*) was used as follows: 0–1 min, 2% B/D; 1–9 min, 2–50% B/D; 9–14 min, 50–98% B/D; 14–15 min, 98% B/D; 15–15.5 min, 98–2% B/D; 15.5–17 min, 2%B/D. For MS, a Thermo Q Exactive Focus mass spectrometer (Thermo Fisher Scientific, Waltham, MA, USA) was set to an ESI mode with sMRM scanning. The spray voltage was 3.8 kV and −2.5 kV in positive and negative modes, respectively. The ion source temperature was set at 400 °C. The analyzer scanned over a mass range of *m*/*z* 81–1000 for a full scan at a mass resolution of 70,000. Data-dependent acquisition (DDA) MS/MS experiments were performed with an HCD scan. The normalized collision energy was 30 eV. Dynamic exclusion was implemented to remove some unnecessary information in the MS/MS spectra.

### 4.5. RNA Extraction and RNA-Seq

Since the unigenes were assembled without a reference genome, the gene expression level denoted as FPKM is not reliable. The transcript level of biosynthetic genes of the garlic odor compound pathway was validated by qRT-PCR. Total RNA was extracted using the Trizol method, as previously described [38]. A total amount of 1 μg of RNA per sample was used as input material for the RNA sample preparations. The purity, concentration, and integrity of the RNA samples were examined by NanoDrop, Qubit 2.0, Agilent 2100 (Agilent Technologies, de novo, Santa Clara, CA, USA). Qualified RNA was processed for library construction and Illumina sequencing was carried out by Biomarker (Beijing, China). Clean data were obtained after trimming adapter contaminations and removing nucleotides with low-quality scores. Clean reads were mapped to the garlic genome (Garlic V2 assembly, PRJNA606385) by using an alignment tool with high efficiency (HISAT2) [39]. Alternatively, unigenes were assembled without a reference genome by using Trinity [40]. Unigenes were annotated by BLAST against such databases as NR, Swiss-Prot, COG, KOG, and KEGG [41]. Amino acid sequences were searched against Pfam databases by using HMMER [42]. Reads from clean data were then mapped to unigenes, and only the mapped reads were used in the following analysis. Fragments per kilobase of transcript, per million mapped reads (FPKM) was used to measure the expression level of an unigene by using Bowtie and RSEM [43,44].

### 4.6. Gene Sequence Cloning and qRT-PCR

Total RNA was extracted from leaves and roots by using TaKaRaMiniBEST Plant RNA Extraction Kit (Takara, Japan). cDNA was synthesized by using the PrimeScript™ RT reagent Kit (Perfect Real Time) (Takara, Japan). qRT-PCR was done by using TB Green^®^ *Premix Ex Taq*™ II (TliRNaseH Plus) (Takara, Japan) and CFX96 Real-Time PCR detection systems (Bio-Rad, Hercules, CA, USA). Primers for gene sequence cloning were designed according to unigenes identified from transcriptomes (Table S5); PCR products were purified by a Takara MiniBEST DNA Fragment Purification Kit (Takara, Japan) and sequenced by Sangon Biotech (Shanghai) Co. Primers for qRT-PCR were designed according to the final sequences corrected by PCR-method (Table S5). Sequences of candidate inner reference genes *EF-1α*, *18SrRNA*, and *Actin* were downloaded following a study of selection and validation of garlic reference genes for qRT-PCR [45], but new primers were designed based on our assembled transcript sequence. The stability of these candidate inner reference genes were evaluated by the BestKeeper tool, which is based on the standard deviation (SD) and the coefficient of variation (CV) calculated with the Ct values of all candidate reference genes [46]. Reference genes with the highest SD and CV values are considered as the least stable genes. Amplification efficiency (E, E = 10[−1/slope] − 1) of all the newly designed primers in qRT-PCR were calculated by utilizing standard curves with ten-fold serial dilutions (10, 10^2^, 10^3^, 10^4^, and 10^5^ dilutions) of each cDNA template. Those primers with an E-value ranging 90–110% were taken as high PCR-amplification efficiency. Following qRT-PCR data collection, Ct values were converted to relative quantities using the formula: 2^−ΔCt^, where ΔCt is the target gene Ct value minus the reference gene Ct value.

### 4.7. Statistical Analysis, Data Processing, and Figure Presentation

The experiments of metabolite detection were repeated 6 times for each sample, while other experiments including RNA-seq were repeated 3 times for each sample. The significant difference of physiological indices was analyzed using the IBM SPSS Statistics 22 (IBM Co., New York, NY, USA). Differential accumulated metabolites (DAMs) were identified by Student’s *t*-test (*p*-value ≤ 0.05). The DAMs were subjected to pathway analysis using the MetaboAnalysis 4.0 database (http://www.metaboanalyst.ca/, accessed on 10 May 2019). Pathway impact factor was used to quantify the enrichment of a pathway.

Differential expressed genes (DEGs) were identified by DESeq2 [47], and the criteria for differentially expressed genes was set as Fold Change (FC) ≥ 4 and FDR < 0.01. Gene functional annotation and pathway enrichment analysis of DEGs were analyzed by using the Kyoto Encyclopedia of Genes and Genomes (KEGG) and gene ontology (GO). The GO terms and pathways with q value (adjusted *p*-value by BH method) < 0.05 were considered to be significantly enriched ones. The enrichment factor = (number of pathway DEGs/total number of all DEGs number)/(number of pathway genes/total number of all genes).

## Figures and Tables

**Table 1:** Physiological comparison between Chinese chives and wild Chinese chives.

	Tuberosum	WL Hookeri	EWL Hookeri	Funckiaefolium
Dry matter (mg/gFW)	67.09 ± 2.09 c	95.01 ± 4.44 b	96.75 ± 3.74 b	105.52 ± 3.86 a
Soluble sugar (mg/gFW)	10.87 ± 0.95 c	15.49 ± 0.29 a	14.79 ± 0.36 b	10.49 ± 0.75 c
Vitamin C (mg/gFW)	0.23 ± 0.02 ab	0.21 ± 0.01 b	0.23 ± 0.01 ab	0.24 ± 0.02 a
Soluble protein (mg/gFW)	2.37 ± 0.10 c	2.97 ± 0.05 a	2.94 ± 0.03 a	2.88 ± 0.03 b

The different letters following the numbers indicate significant differences (*p* < 0.05) in Duncan’s multiple range test.

**Table 2:** Top 20 GO terms in GO enrichment analysis of DEGs.

GO ID	GO Term	Annotated	DEG N	Expected N	*p*-KS
GO:0015979	photosynthesis	240	205	185.14	4.3 × 10^−12^
GO:0009658	chloroplast organization	81	76	62.49	1.1 × 10^−8^
GO:0018298	protein-chromophore linkage	62	52	47.83	9.9 × 10^−8^
GO:0009765	photosynthesis, light harvesting	59	50	45.51	1.5 × 10^−7^
GO:0055114	oxidation-reduction process	1986	1489	1532.04	1.5 × 10^−5^
GO:0016117	carotenoid biosynthetic process	26	26	20.06	1.6 × 10^−5^
GO:0009793	embryo development ending in seed dormancy	153	135	118.03	2.1 × 10^−5^
GO:0000413	protein peptidyl-prolyl isomerization	91	82	70.2	2.5 × 10^−5^
GO:0006418	tRNA aminoacylation for protein translation	107	88	82.54	3.8 × 10^−5^
GO:0010027	thylakoid membrane organization	22	21	16.97	5.4 × 10^−5^
GO:0046835	carbohydrate phosphorylation	48	42	37.03	1.4 × 10^−4^
GO:0010207	photosystem II assembly	19	19	14.66	2.0 × 10^−4^
GO:0006400	tRNA modification	81	74	62.49	4.2 × 10^−4^
GO:0006000	fructose metabolic process	6	6	4.63	5.2 × 10^−4^
GO:0006003	fructose 2,6-bisphosphate metabolic process	6	6	4.63	5.2 × 10^−4^
GO:0048366	leaf development	88	79	67.89	6.0 × 10^−4^
GO:1901566	organonitrogen compound biosynthetic process	877	689	676.54	6.6 × 10^−4^
GO:0001731	formation of translation preinitiation complex	41	32	31.63	7.2 × 10^−4^
GO:0009814	defense response, incompatible interaction	38	35	29.31	7.7 × 10^−4^
GO:0009657	plastid organization	111	105	85.63	8.5 × 10^−4^

DEG N, number of DEGs enriched; Expected N, expected number enriched; *p*-KS, *p*-value of KS test.

**Table 3:** Sequence information of biosynthetic genes of garlic odor compounds.

	Length of AA ^1^	Identity with Transcript	Identity with NCBI Database ^2^
*AhGGT1*	593	99.1%	90.35%; BAQ21911.1
*AhGGT1E*	593	99.3%	89.92%; BAQ21911.1
*AtGGT1*	629	99.8%	92.24%; BAQ21911.1
*AhGGT2*	622	99.5%	87.24%; BAQ21912.1
*AhGGT2E*	622	100%	88.27%; BAQ21912.1
*AtGGT2*	622	99.5%	89.23%; BAQ21912.1
*AhGGT3*	626	98.9%	86.92%; BAQ21913.1
*AhGGT3E*	626	98.9%	87.20%; BAQ21913.1
*AtGGT3*	584	99.3%	86.11%; BAQ21913.1
*AhPCS1*	505	98.8%	89.35%; AAO13809.1
*AhPCS1E*	505	97.7%	89.16%; AAO13809.1
*AtPCS1*	505	98.8%	92.49%; AAO13809.1
*AhFMO1*	458	99.4%	87.00%; 6WPU_A
*AhFMO1E*	317	92.4%	88.73%; 6WPU_A
*AtFMO1*	462	97.5%	91.88%; 6WPU_A
*AhALN*	471	91.9%	91.15%; AYN25508.1
*AhALNE*	471	91.7%	90.88%; AYN25510.1
*AtALN*	471	100%	100%; BAA20358.1

^1^ Length of amino acid sequence. ^2^ Sequences in the database that show the highest identity with the query sequences. AYN25508.1 was derived from *Allium chinense*; AYN25510.1 and BAA20358.1 were derived from *A. tuberosum*; the rest of the sequences were from *A. sativum*.

**Table 4:** Stability comparison of the candidate genes analyzed and ranked by BestKeeper.

Group	Rank	Gene	SD	CV
Leaf	1	*EF-1α*	0.37	2.07
	2	*18SrRNA*	0.57	5.44
	3	*Actin*	1.27	5.92
Bulb	1	*EF-1α*	0.58	3.35
	2	*18SrRNA*	1.12	11.02
	3	*Actin*	1.32	6.16
Root	1	*18SrRNA*	0.74	7.75
	2	*EF-1α*	0.80	4.78
	3	*Actin*	0.89	4.23

## Data Availability

Data are contained within the article or Supplementary Materials. Data of RNA-seq were deposited in NCBI: reference number (PRJNA832428). Gene sequences of garlic odor compound biosynthesis can be found in supplementary tables.

## Supplementary Materials

The following supporting information can be downloaded at: https://www.mdpi.com/article/10.3390/ijms23137013/s1.
