# Supplementary material for: Biosynthesis and Metabolism of Garlic Odor Compounds in Cultivated Chinese Chives (Allium tuberosum) and Wild Chinese Chives (Allium hookeri)
Source: Int J Mol Sci. 2022 Jun 24;23(13):7013. doi: 10.3390/ijms23137013 (PMC9266804; doi:10.3390/ijms23137013)
Supplement: Supplementary file 1 [file ijms-23-07013-s001.zip › supplementary figures.pdf]

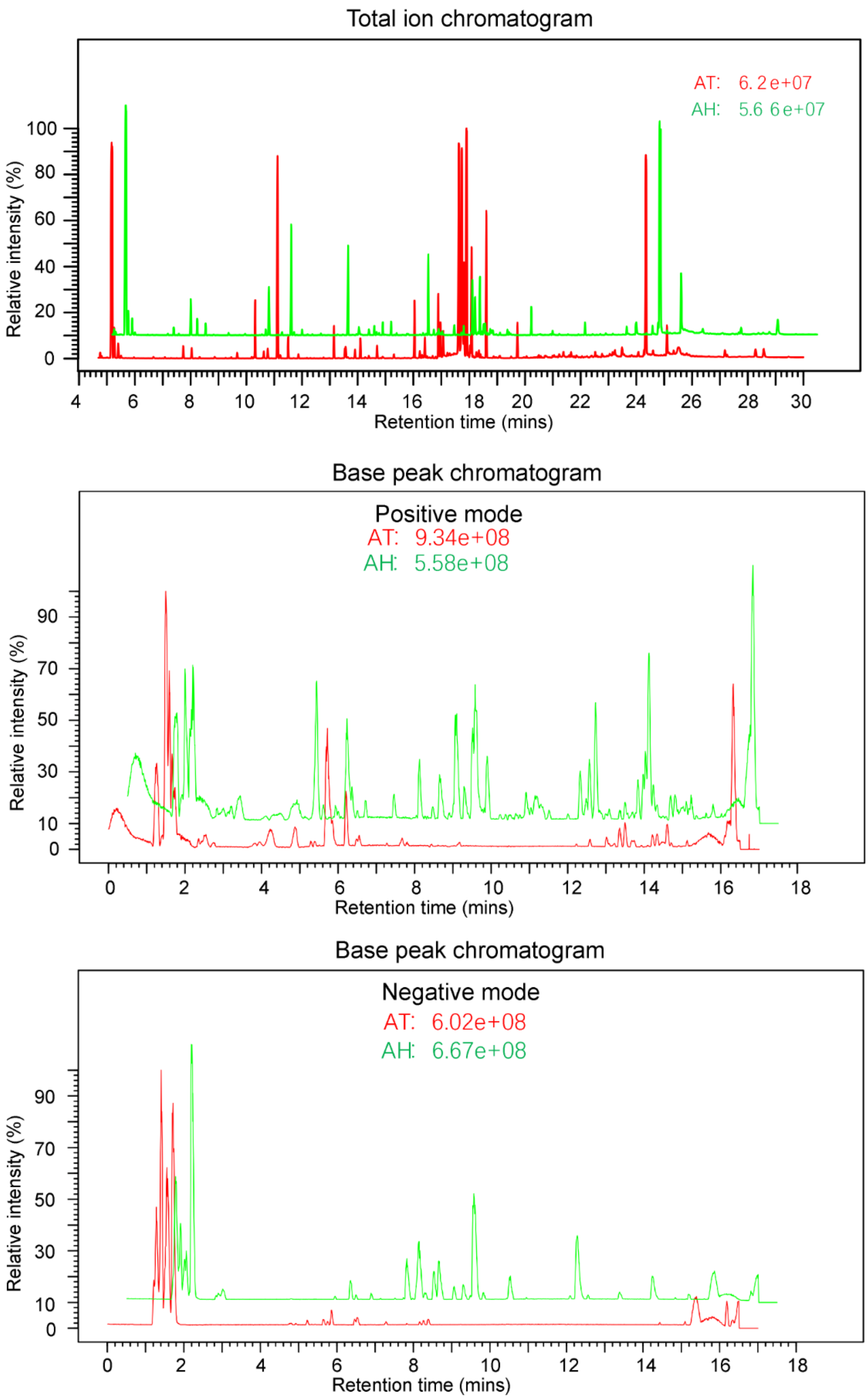

Figure S1 Chromatogram pictures. Total ion chromatogram in GC-MS; base peak chromatogram under positive mode and under negative mode in LC-MS. Red lines are for samples of AT (*A. tuberosum*); Green lines are for samples of AH (*A. hookeri*).

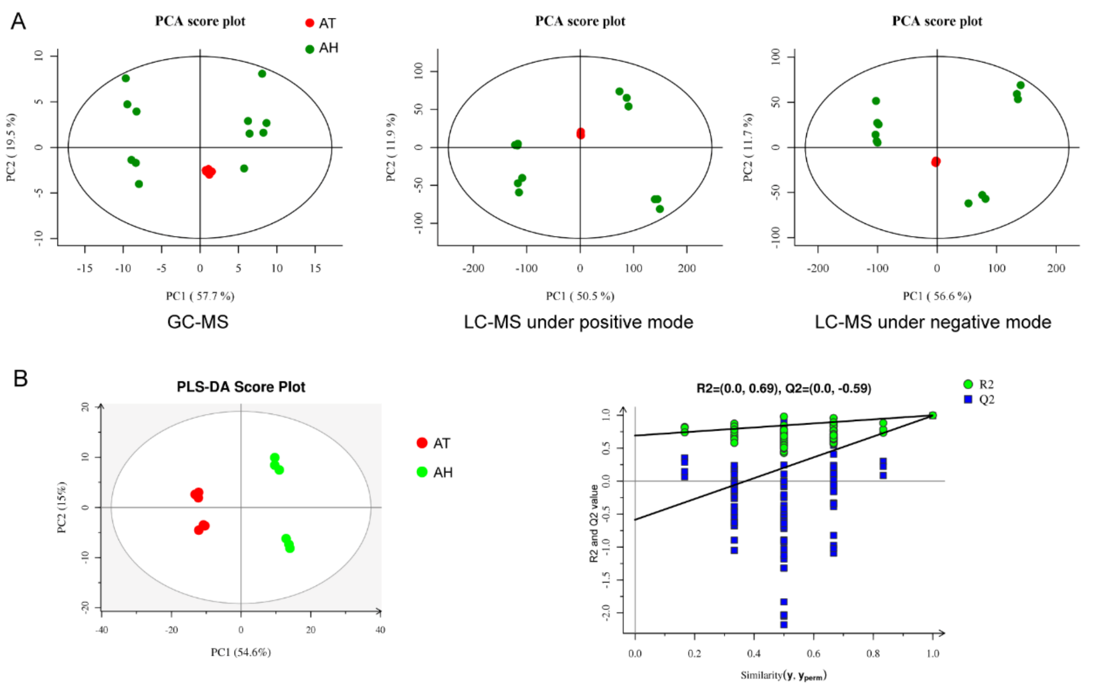

Figure S2 Quality control and quality assurance by two dimensionality-reduction statistical methods. (A) Principle component analysis (PCA), an unsupervised analysis, was applied to metabolites detected in GC-MS and LC-MS, separately. Red dots indicate samples of AT (*A. tuberosum*); green dots indicate samples of AH (*A. hookeri*). (B) Partial least squares-discriminate analysis (PLS-DA), a supervised analysis, was applied to all the detected metabolites. R2, goodness of fit or explained variation; Q2, goodness of prediction or predicted variation. Q2 is the R2 when the PLS built on a training set is applied to a test set. In the analysis, R2X=0.696 (>0.5), R2Y=0.998 (>0.5), Q2=0.992 (>0.5), indicate high predictive accuracy. Left panel, PLS-DA score plot. Red dots indicate samples of AT; green dots indicate samples of AH. Right panel, permutations plot indicates there is no overfitting problem in PLS-DA since Q2=-0.59 (<0) on y-axis in the linear regression analysis of Q2 value.

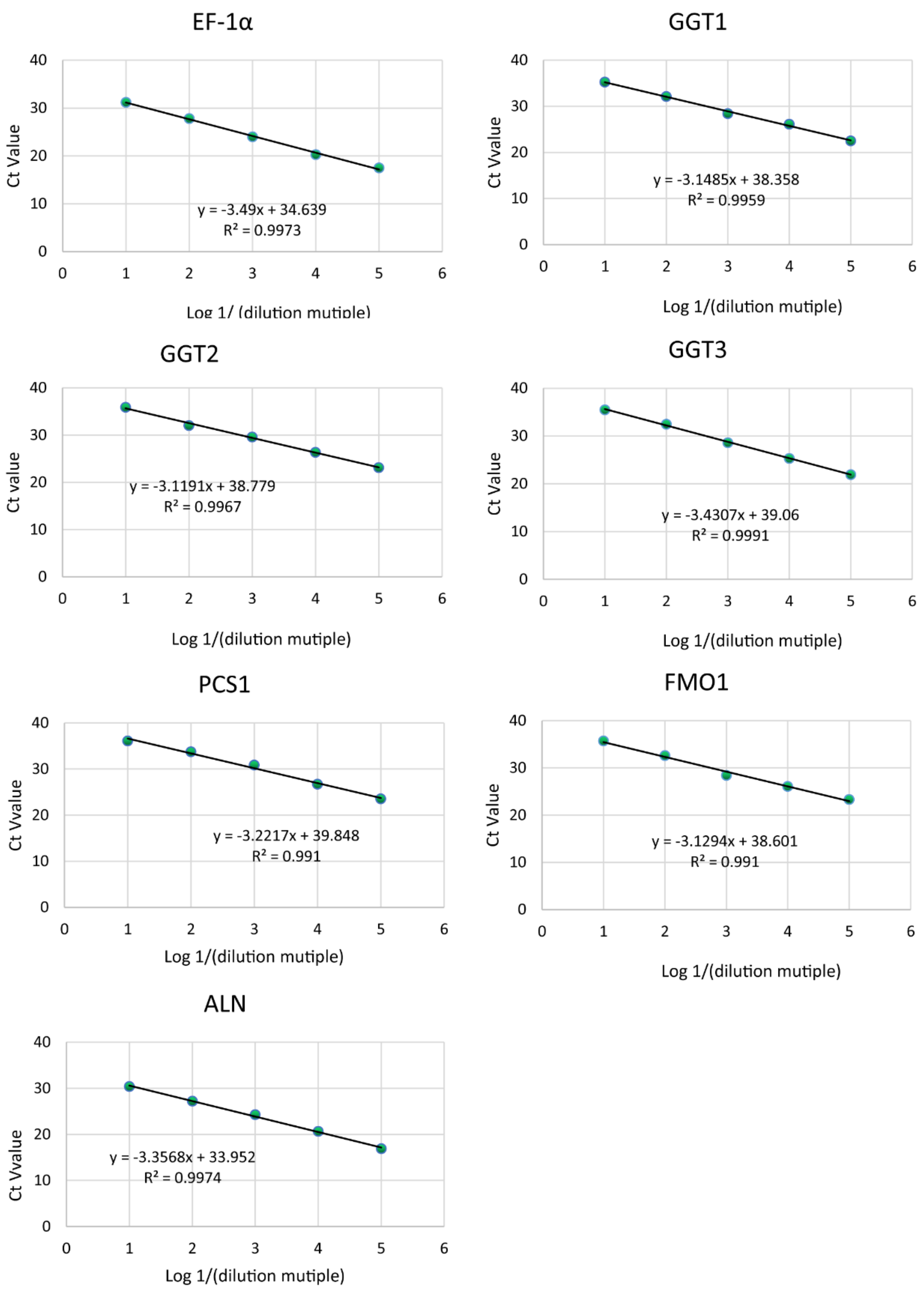

Figure S3 Standard curves showing the PCR efficiency values for the reference genes and the six target genes, generated from a 10- fold dilution series of cDNA. EF-1α with an E=93.43%, R^2=0.9973. GGT1 with an E=107.78%, R^2=0.9959. GGT2 with an E=109.22%, R^2=0.9967. GGT3 with an E=95.65%, R^2=0.9991. PCS1 with an E=104.36%, R^2=0.991. FMO1 with an E=108.71%, R^2=0.991. ALN with an E=98.57%, R^2=0.9974.
